# Manifestaciones orales del virus de la chikungunya. un estudio transversal

**DOI:** 10.21142/2523-2754-1302-2025-241

**Published:** 2025-05-16

**Authors:** María del Carmen González Galván, José Gamarra Insfrán, Carlos González Casamada

**Affiliations:** 1 Facultad de Odontología, Cátedra de Patología Bucal, Universidad Nacional de Asunción. Asunción, Paraguay. maricarmenggalvan@gmail.com, josemgamarra31@gmail.com Universidad Nacional de Asunción Facultad de Odontología, Cátedra de Patología Bucal Universidad Nacional de Asunción Asunción Paraguay maricarmenggalvan@gmail.com josemgamarra31@gmail.com; 2 Facultad de Odontología, Cátedra de Metodología de la Investigación, Universidad Nacional de Asunción. Asunción, Paraguay, casamadacarlosraul@gmail.com Universidad Nacional de Asunción Facultad de Odontología, Cátedra de Metodología de la Investigación Universidad Nacional de Asunción Asunción Paraguay casamadacarlosraul@gmail.com

**Keywords:** virus chikungunya, salud oral, salud pública (MeSH), chikungunya virus, oral health, public health (MeSH)

## Abstract

**Introducción:**

: El virus chikungunya se aisló en humanos por primera vez en 1952, tras una epidemia en Tanzania. Durante 2023 ocurrió la mayor epidemia del chikungunya en Paraguay. Objetivo: Determinar las manifestaciones orales más frecuentes autorreportadas por pacientes infectados por el chikungunya en un grupo de la población paraguaya.

**Materiales y métodos:**

Se realizó un estudio observacional transversal, durante los meses de septiembre y octubre de 2024, con sujetos de investigación que padecieron chikungunya. El instrumento contó con dos secciones: la primera abarcó la información del estudio y los datos sociodemográficos, mientras que en la segunda se indicó la presencia de alguna manifestación oral durante el trascurso de la enfermedad. Las opciones estuvieron catalogadas de manera dicotómica (“sí” o “no”) y los sujetos que indicaron la presencia de alguna manifestación oral también indicaron su intensidad (“leve”, “moderada” o “severa”).

**Resultados:**

Participaron 131 sujetos, entre los que predominó el sexo femenino, y el promedio de edad fue de 35 años. Las tres manifestaciones orales más frecuentes fueron el dolor de la encía (43,5%), la sensación de ardor en la boca (41,9%) y la presencia de úlceras (31,2%). Las úlceras orales fueron consideradas como la manifestación más severa.

**Conclusión:**

Las manifestaciones orales reportadas con más frecuencia fueron el dolor de la encía, la sensación de ardor y la presencia de úlceras orales. En cuanto a la intensidad, las manifestaciones orales más severas fueron la presencia de úlceras, la dificultad para masticar o tragar y la pérdida del gusto.

## Abstract (en)

**Introduction:**

chikungunya virus was isolated in humans for the first time in 1952 after an epidemic in Tanzania, during 2023 the largest chikungunya epidemic occurred in Paraguay.

**Objective:**

to determine the most frequent oral manifestations self-reported by chikungunya-infected patients in a Paraguayan population group. Materials and methods: a cross-sectional observational study was carried out during September and October 2024, with research subjects who suffered from chikungunya. The instrument had two sections: the first covered the study information and sociodemographic data. The second section indicated the presence of any oral manifestation during the course of the disease, the options were catalogued in a dichotomous manner (“yes” or “no”), the subjects who indicated the presence of any oral manifestation also indicated its intensity (“mild”, “moderate” or “severe”).

**Results:**

131 subjects participated; female sex predominated and the average age was 35 years. The three most frequent oral manifestations were gum pain (43.5%), burning sensation in the mouth (41.9%) and the presence of ulcers (31.2%). Oral ulcers were considered the most severe manifestation.

**Conclusion:**

the most frequently reported oral manifestations were gum pain, burning sensation and the presence of oral ulcers. In terms of intensity, the most severe oral manifestations were the presence of ulcers, difficulty in chewing and/or swallowing and loss of taste.

## INTRODUCCIÓN

El virus de la chikungunya (CHIKV) fue reconocido por primera vez como patógeno humano en África durante la década de 1950. La fiebre chikungunya se considera un arbovirus de aparición brusca que se transmite a través de mosquitos. El *Aedes aegypti* y el *Aedes albopictus* son los principales hospedadores intermediarios del CHIKV ^1^^,^^2^. La distribución geográfica del *Aedes aegypti* en América del Sur se ha ampliado en las últimas décadas y el área de distribución del *Aedes albopictus* abarca diecinueve países en este continente, entre los que se encuentra Paraguay ^3^^, 4)^.

El periodo de incubación del CHIKV va de dos a cuatro días y, tras él, se produce el periodo agudo de la enfermedad. Este periodo puede evolucionar a una forma subaguda (quince a noventa días) y crónica (más de noventa días), y ya se ha demostrado que más del 40% de los pacientes evolucionan a la forma crónica. La calidad de vida de los pacientes con la fiebre del chikungunya se deteriora considerablemente durante las diferentes fases de la enfermedad ^5^^-^^7^.

Los primeros síntomas abarcan fiebre, debilidad, dolores articulares y erupciones cutáneas. Cuando los síntomas persisten, se manifiestan en forma de poliartritis constante o poliartralgia acompañada de rigidez matutina y astenia, que puede durar desde meses hasta más de tres años desde el inicio de la infección. En pacientes con CHIKV, la prevalencia de las distintas manifestaciones orales es muy variable; va desde el 20 hasta el 95% ^8^^-^^10^. Las lesiones orales pueden localizarse en los labios, la lengua, el suelo de la boca, el paladar, las encías, la mucosa yugal, entre otras zonas. La presencia de úlceras, eritemas, ardor en la mucosa y dificultad para abrir la boca son algunas de las manifestaciones detectadas con mayor frecuencia ^11^^,^^12^.

Evidencia científica reciente establece la patogenia y la epidemiología del CHIKV en diversos puntos geográficos; sin embargo, la evidencia que reporte las manifestaciones orales más frecuentes de este virus es escasa. Teniendo en cuenta que durante el verano de 2023 ocurrió la mayor epidemia de chikungunya en Paraguay ^13^ y la inexistencia de un manual de procedimientos clínicos para el odontólogo ante la aparición de estas manifestaciones, el objetivo de la presente investigación es determinar las manifestaciones orales más frecuentes autorreportadas por un sector de la población paraguaya durante su infección por el CHIKV. A partir de la obtención de estos datos se podrían potenciar, mediante conferencias y materiales didácticos, los conocimientos teóricos y prácticos que tengan como finalidad la resolución de estas manifestaciones orales y, de esa manera, mejorar los servicios brindados a la población afectada por este virus.

## MATERIALES Y MÉTODOS

Se realizó un estudio observacional descriptivo de corte transversal, con una muestra conformada por 131 sujetos que padecieron la fiebre del CHIKV, reclutados a través de un muestreo no probabilístico por bola de nieve, durante los meses de septiembre y octubre de 2024. El protocolo de investigación (P028-2023) fue aprobado por el Comité de Ética en Investigación de la Facultad de Odontología de la Universidad Nacional de Asunción.

El instrumento de medición se elaboró con base al trabajo de Katti *et al*. ^14^ en el que se evaluó clínicamente la cavidad oral de pacientes con el virus del CHIKV. Se aplicó un cuestionario utilizando la herramienta Formularios de Google, distribuida a través de un enlace por la aplicación WhatsApp.

El cuestionario contó con dos secciones. En la primera, las personas recibieron información del estudio, podían consentir participar voluntariamente del mismo y luego debían aportar algunos datos sociodemográficos como edad, sexo y la presencia de enfermedades de base. En la segunda sección, debían indicar si presentaron alguna manifestación oral durante el trascurso de la enfermedad, como dolor de encía, sangrado de la encía, sensación de ardor en la boca, dificultad para abrir la boca, dificultad para masticar y/o tragar, presencia de úlceras, halitosis, salivación excesiva, pérdida del gusto y movilidad excesiva de los dientes. Las opciones de respuestas estaban catalogadas de manera dicotómica como “sí” o “no”. Si el paciente indicaba la presencia de alguna manifestación, también debía indicar la intensidad, ya sea “leve”, “moderada” o “severa”. Al finalizar el cuestionario se proporcionó un enlace para la descarga de una infografía con información relevante sobre el CHIKV.

En relación con los datos sociodemográficos, la edad fue considerada como una variable cuantitativa y se utilizó una escala de medición discreta; para el sexo, variable cualitativa, se utilizó una escala dicotómica. Todas las manifestaciones orales (dolor de encías, sangrado de encías, sensación de ardor en la boca, incapacidad para masticar y/o tragar, halitosis, ulceración, dificultad/dolor al abrir la boca, salivación excesiva, pérdida del gusto, aflojamiento de dientes), los diferentes niveles de intensidad (leve, moderado, severo) de las manifestaciones orales y las enfermedades de base (diabetes, hipo e hipertensión arterial, enfermedades cardíacas, artritis y lupus) han sido considerados como variables cuantitativas.

### Análisis estadístico

Todos los datos fueron trasladados a una planilla electrónica utilizando Excel Office 365. Para el análisis estadístico, se realizó un análisis descriptivo mediante frecuencias y porcentajes con los *softwares* R y Epi Info 7.0. Para evaluar la asociación entre variables categóricas, se empleó la prueba de chi-cuadrado (χ²), mientras que para la comparación de medias entre dos grupos independientes se utilizó la prueba t de Student para muestras independientes. Se consideró un nivel de significancia de p < 0,05 para todas las pruebas estadísticas.

## RESULTADOS

Un total de 131 sujetos participaron del estudio. La edad media de los participantes fue de 35,3 ± 14,5, con un rango de 18 a 71 años (Tabla 1). Se observó una significancia estadística entre la presencia de manifestaciones orales con el sexo femenino (p < 0,001). En relación con la presencia de enfermedades de base, solo el 25,1% de los encuestados refirió padecer alguna y no se observó una asociación significativa de esta variable con la edad ni con el sexo (p > 0,05).

**Tabla 1:** Datos sociodemográficos de un sector de la población paraguaya afectada por el CHIKV

Variable	Porcentaje
Rango de edad	
18-19	3,8
20-29	40,4
30-39	22,2
40-49	16,8
≥50	16,8
Sexo	
Femenino	80,9
Masculino	19,1

Los participantes reportaron poseer más de una manifestación oral durante el padecimiento de la infección por el CHIKV. El 43,5% manifestó dolor de la encía seguida por la sensación de ardor en la boca (41,9%), siendo estas las manifestaciones orales más frecuentes; por el contrario, la hipersalivación y la movilidad de los dientes fueron las manifestaciones menos frecuentes en un 14,5 y un 9,1% respectivamente (Tabla 2). 

**Tabla 2:** Frecuencia de las manifestaciones orales reportadas por un sector de la población paraguaya durante la infección del CHIKV

Manifestaciones	n.º	Porcentaje
Dolor de la encía	57	43,5
Ardor en la boca	55	41,9
Presencia de úlceras	41	31,2
Dificultad para masticar/tragar	40	30,5
Halitosis	38	29,0
Pérdida del gusto	38	29,0
Sangrado de la encía	37	28,2
Dificultad para abrir la boca	36	27,4
Salivación excesiva	19	14,5
Movilidad de los dientes	12	9,1

En el análisis estadístico que relacionó a las manifestaciones orales con la edad, se identificó una relación significativa con el dolor de encías (p = 0,010), el sangrado de encías (p = 0,002) y la sensación de ardor (p = 0,002), lo que sugiere que estas manifestaciones pueden estar influenciadas por la edad de los sujetos. En cuanto al análisis estadístico que relacionó las manifestaciones orales con el sexo, se observaron asociaciones significativas con el dolor de encías (p = 0,001) y con el sangrado de encías (p = 0,031). No se encontraron diferencias estadísticamente significativas en las demás manifestaciones orales (p > 0,05).

Con respecto a la intensidad de las manifestaciones orales, las úlceras y la incapacidad para masticar/tragar fueron las más severas, con un 36,7 y un 36,4%, respectivamente. El dolor de la encía y el ardor en la boca, las manifestaciones orales más frecuentes, fueron reportadas con intensidad leve (Tabla 3). No se observaron significancias estadísticas entre las manifestaciones orales y su intensidad (p > 0,05).

**Tabla 3:** Intensidad de las manifestaciones orales reportadas por un sector de la población paraguaya durante la infección del CHIKV

Manifestaciones	Leve	Moderada	Severa
Presencia de úlceras	30,6	32,7	36,7
Dificultad al masticar/tragar	20,5	43,2	36,4
Pérdida del gusto	24,3	48,6	27,0
Dificultad para abrir la boca	26,2	47,6	26,2
Dolor en la encía	45,3	34,4	20,3
Ardor en la boca	48,3	33,3	18,3
Movilidad de dientes	50,0	33,3	16,7
Halitosis	48,8	37,2	14,0
Salivación excesiva	53,8	34,6	11,5
Sangrado de la encía	44,2	48,8	7,0

## DISCUSIÓN

El presente estudio se llevó a cabo a partir del autorreporte de las manifestaciones orales desarrolladas por un grupo de paraguayos infectados por el CHIKV. La mayoría de los estudios han evaluado las manifestaciones sistémicas de la chikungunya, pero pocos han reportado las manifestaciones orales de la enfermedad. Existe un reporte realizado en Paraguay acerca del CHIKV, pero este se centra en las lesiones dermatológicas atípicas ^15^.

Los resultados de la presente investigación arrojaron que las manifestaciones más frecuentes fueron el dolor de encías, la sensación de ardor en la boca y la presencia de úlceras orales. Desde un panorama general, esto coincide con las manifestaciones principales reportadas en un estudio realizado en Brasil, en los que los casos de infección por el virus chikungunya han notificado dolor, ardor y sangrado en las encías, incapacidad para deglutir y masticar, entre otras ^16^. Con relación a la intensidad, las úlceras orales fueron reportadas como la manifestación más severa, seguidas por la dificultad para masticar/tragar los alimentos y la pérdida del gusto. En cuanto a los datos sociodemográficos, se observó una significancia estadística entre la presencia de manifestaciones orales y el sexo femenino, lo que concuerda con resultados obtenidos en India, Sri Lanka y Colombia, en los que el riesgo relativo de padecer CHIKV en las mujeres fue superior al de los hombres ^12^^,^^17^^,^^18^.

El 28% de los participantes del presente estudio reportó sangrado de encías. En trabajos similares ^11^^,^^14^, esta manifestación oral se observó en los participantes con distintos porcentajes de prevalencia (del 33% al 55%), por lo cual la población paraguaya fue la que presentó menor sangrado de encía. El 31% de los participantes de este estudio manifestó presentar úlceras orales, lo que supera los resultados obtenidos en trabajos originarios de Brasil e India ^11^^,^^14^. En lo que concierne a la halitosis, se observó en el 29% de los participantes de este estudio. En una población india, esta manifestación oral fue detectada en el 21% de los sujetos. Además de los porcentajes cercanos, se destaca que el tamaño de las muestras en ambos estudios es similar.

La presencia de manifestaciones orales puede condicionar la alimentación de los pacientes al limitar la dieta a alimentos blandos o semisólidos, e incluso a una dieta líquida en los casos más severos ^19^. Al respecto, un estudio brasileño reportó que el 95% de los participantes presentó odinofagia y/o disfagia ^11^, lo que supera ampliamente al 30% de los participantes paraguayos que reportó dificultad para masticar o tragar. Esta diferencia tan significativa podría deberse a la terminología y los conceptos utilizados al elaborar las encuestas. En un reporte procedente de Pakistán, la dificultad para abrir la boca fue la manifestación oral más común encontrada entre los pacientes de 45 a 60 años ^20^. Este dato difiere de los resultados de la presente investigación, en la cual la dificultad para abrir la boca se manifestó en el 27% de los participantes; por lo cual no fue considerada entre las más frecuentes.

En cuanto a las limitaciones del estudio, se debe mencionar que, al ser un cuestionario autoadministrado, pueden existir dificultades en las interpretaciones de las preguntas por parte de los sujetos de investigación. Con la intención de reducir los sesgos debido a este factor, para la redacción de la encuesta se utilizó un lenguaje coloquial. También se debe mencionar que no se realizaron evaluaciones clínicas odontológicas ni análisis serológicos a los sujetos de investigación, lo que implica que el estudio se basó exclusivamente en el relato proporcionado por los sujetos de investigación. Estas limitaciones se deben principalmente al diseño del estudio y a su carácter virtual, lo que debería ser considerado por futuros estudios.

A pesar de las limitaciones mencionadas, este informe recolecta las manifestaciones orales más frecuentes y las clasifica de acuerdo con su intensidad. Además, podría considerarse el primero en abarcar el tema en el territorio paraguayo. Otros estudios incluyeron varias manifestaciones orales distintas a las abarcadas en este informe; estas podrían ser de utilidad para futuras investigaciones. Las lesiones orales pueden dificultar o impedir la adecuada higiene oral. Lo dicho, sumado a los síntomas sistémicos del chikungunya, implica un deterioro considerable de la calidad de vida de los pacientes. Por tanto, continuar con trabajos que integren a profesionales de salud de distintas disciplinas y que fortalezcan la línea de investigación será de gran importancia para la generación de conocimientos más amplios y que estos favorezcan la recuperación integral de los pacientes afectados por esta patología sistémica que repercute en la salud oral.

## CONCLUSIÓN

Las manifestaciones orales reportadas con más frecuencia fueron el dolor de la encía, la sensación de ardor en la boca y la presencia de úlceras orales. Las manifestaciones orales reportadas como las más severas fueron la presencia de úlceras, la dificultad para masticar y/o tragar, y la pérdida del gusto.

## Financiamiento:

Autofinanciado
